# Entorhinal Cortex Atrophy in Early, Drug-naive Parkinson’s Disease with Mild Cognitive Impairment

**DOI:** 10.14336/AD.2018.1116

**Published:** 2019-12-01

**Authors:** Xiuqin Jia, Zhijiang Wang, Tao Yang, Ying Li, Shuai Gao, Guorong Wu, Tao Jiang, Peipeng Liang

**Affiliations:** ^1^Department of Radiology, Beijing Chaoyang Hospital, Capital Medical University, Beijing, China.; ^2^Institute of Mental Health, Peking University Sixth Hospital, Beijing 100191, China.; ^3^National Clinical Research Center for Mental Disorders and Key Laboratory of Mental Health, Ministry of Health, Peking University, Beijing, China.; ^4^Beijing Municipal Key Lab for Translational Research on Diagnosis and Treatment of Dementia, Beijing, China.; ^5^Department of Psychology, Tsinghua University, Beijing, China.; ^6^Department of Radiology, Beijing Anzhen Hospital, Capital Medical University, Beijing, China.; ^7^Department of Radiology and BRIC, University of North Carolina at Chapel Hill, Chapel Hill, NC 27599, USA.; ^8^School of Psychology, Capital Normal University, Beijing, China.

**Keywords:** Parkinson's disease, mild cognitive impairment, voxel-based morphometry, entorhinal cortex, amygdala

## Abstract

Patients with Parkinson’s disease (PD) generally have a higher proportion of suffering from mild cognitive impairment (MCI) than normal aged adults. This study aimed to identify the specific neuroanatomical alterations in early, drug-naive PD with MCI (PD-MCI) by comparing to those PD with normal cognition (PD-NC) and healthy controls (HCs), which could help to elucidate the underlying neuropathology and facilitate the development of early therapeutic strategies for treating this disease. Structural MRI data of 237 early, drug-naive non-demented PD patients (classified as 61 PD-MCI and 176 PD-NC) and 69 HCs were included from Parkinson's Progression Markers Initiative (PPMI) database after data quality control. Within these data, a subset of 61 HCs and a subset of 61 PD-NC who were matched to the 61 PD-MCI group for age, gender, and education-level were selected to further eliminate the sample size effect. The gray matter (GM) volume changes between groups were analyzed using voxel-based morphometry (VBM). Furthermore, correlations between GM volume alterations and neuropsychological performances and non-cognitive assessments (including olfactory performance) were further examined. Compared to HC, patients with PD-NC and PD-MCI commonly exhibited atrophies in the bilateral amygdala (AM) and the left primary motor cortex (M1). Patients with PD-MCI exclusively exhibited atrophy in the right entorhinal cortex (ENT) compared to PD-NC. Significantly negative correlations were found between GM loss in the bilateral AM and olfactory performance in all PD patients, and between ENT loss and memory performance in PD-MCI. The findings suggest that the right ENT atrophy may subserve as a biomarker in early, drug-naive PD-MCI, which shed light on the neural underpinnings of the disease and provide new evidence on differentiating the neuroanatomical states between PD-MCI and PD-NC.

Mild cognitive impairment (MCI) frequently occurs in patients with Parkinson's disease (PD) and it even exits at the early stage of the disease [1,2]. PD patients with MCI (PD-MCI) are at higher risk of developing dementia compared to patients with normal cognition (PD-NC) [3]. It is thus of great clinical significance to detect neuroanatomical changes that are specific to PD-MCI in order to develop corresponding diagnosis and early treatment to slow its progression to dementia.

Gray matter (GM) volume loss has been reported in PD-MCI using voxel-wise morphometry (VBM). However, these findings still lack consistency. For example, some studies found atrophies in the medial temporal lobe (MTL) [4,5,6,7], and the frontal areas in PD-MCI [8], but others failed to detect any difference between PD-MCI and PD-NC or HC [9,10]. These inconsistences might be due to the heterogeneities of patients, such as different stages of disease [11] and drug-related confounding [12]. The specific structural alterations for early, drug-naive PD-MCI are thus still unclear.

The present study aimed to (1) examine the structural atrophy specific to newly diagnosed, drug-naive PD-MCI patients as compared to those PD-NC and HC, and (2) explore the associations between the GM loss and neuropsychological measures in these patients. According to the pathological changes of PD, the cortical involvement starts from the medial temporal mesocortex, and then spreads to the neocortex (i.e., the frontal cortex) during the course of the disease [13]. It was hypothesized that the early PD-MCI might exhibit GM alterations in the MTL than the frontal cortex.

## MATERILAS AND METHODS

### Subjects

Data used in this study were from Parkinson's progression markers initiative (PPMI) (http://www.ppmi-info.org/, see more details in [14]). Only newly diagnosed, untreated PD patients (*n* = 390) and HCs (*n* = 179) who underwent T1 MRI scanning at baseline were included in the present study.

**Table 1:** Demographic and neuropsychological characteristics.

	HC (*n* = 69)	PD-NC (*n* = 176)	PD-MCI (*n* = 61)	*p*-value
Age (year)	61.78 (6.35)	61.47 (7.81)	64.11 (7.08)	0.088
Gender (m/f)	44/25	107/69	37/24	0.903
Education (year)	16.57 (2.41)	15.77 (2.94)	14.93 (3.14)	0.008*†
TIV	1532.27 (190.25)	1562.93 (142.86)	1559.40 (144.16)	0.371
Hoehn & Yahr stage	—	1.56 (0.51)	1.67 (0.51)	0.148
MDS-UPDRS Part III	—	19.20 (7.81)	22.75 (9.28)	<0.05
Disease duration (month)	—	6.97 (7.15)	7.03 (7.23)	0.954
GDS	0.87 (0.97)	1.46 (1.24)	1.74 (1.17)	<0.001*†
MoCA	28.30 (1.10)	28.11 (1.32)	24.34 (2.11)	<0.001†‡
JoLO	13.41 (1.63)	13.06 (2.02)	12.18 (2.22)	0.002†‡
HVLT-R immediate recall	26.30 (4.16)	25.48 (4.35)	21.56 (5.29)	<0.001†‡
HVLT-R delayed recall	9.36 (2.31)	8.86 (2.22)	7.20 (2.54)	<0.001†‡
LNS	10.97 (2.32)	10.93 (2.58)	9.49 (2.59)	0.001†‡
Semantic fluency total	54.14 (10.69)	50.67 (10.91)	44.26 (10.38)	<0.001†‡
SDMT	49.13 (9.84)	43.60 (8.75)	36.90 (11.38)	<0.001*†‡
UPSIT	36.74 (1.56)	23.28 (8.14)	20.33 (7.82)	<0.001*†‡
SCOPA-AUT	5.09 (2.81)	8.53 (4.74)	10.38 (6.07)	<0.001*†‡

Note: Data are expressed as mean (standard deviation). Gender data were analyzed with χ^2^ test. Other *p* values were derived from Kruskal Wallis test or Mann-Whitney test for non-parametric test and independent one-way ANOVA or two sample *t*-test for parametric test.

* post hoc comparisons showed significant differences between HCs and patients with PD-NC;

† post hoc comparisons showed significant differences between HCs and patients with PD-MCI;

‡ post hoc comparisons showed significant differences between patients with PD-NC and those with PD-MCI. TIV = total intracranial volume; MDS-UPDRS = Movement Disorder Society-Unified Parkinson's Disease Rating Scale; JoLO = Benton Judgement of Line Orientation; GDS = Geriatric Depression Scale; MoCA = Montreal Cognitive Assessment; LNS = Letter-Number Sequencing; HVLT-R = Hopkins Verbal Learning Test-Revised; SDMT = Symbol-Digit Modalities Test; UPSIT = University of Pennsylvania Smell Identification Test; SCOPA-AUT = Scales for Outcomes in Parkinson's disease - Autonomic; HC, healthy control; PD-NC, Parkinson's disease with normal cognition; PD-MCI, Parkinson's disease with mild cognitive impairment.

The selection of the early, drug-naïve non-demented PD patients were required to (1) have at least two of bradykinesia, rigidity, and resting tremor or have either an asymmetric resting tremor or asymmetric bradykinesia; (2) have a recent PD diagnosis (within 2 years) and an early clinical disease stage (Hoehn and Yahr (HY) stage I or II); (3) be untreated; and (4) have a dopamine transporter (DAT) deficit on imaging. Subjects were excluded if they had (1) atypical parkinsonism; (2) a clinical diagnosis of dementia determined by the investigator at each site [15] and a Montreal Cognitive Assessment (MoCA) score less than 22 [16]; (3) categorized as subjects without evidence of dopaminergic deficit (SWEDD) or evolved to other parkinsonism; (4) use of any medication that might interfere with dopamine transporter SPECT imaging; (5) medical or neurologic causes of cognitive impairment (e.g., stroke, brain injury, epilepsy, metabolic abnormalities and major depression); or (6) other PD-associated comorbid conditions that significantly influence cognitive testing. The HCs were required to have no significant neurological dysfunction, no first-degree member with PD, no use of investigational drugs or devices within 60 days before baseline, as well as MoCA score more than 26. For each participating PPMI site, approval from an ethical standards committee on human experimentation was received before study initiation. Written informed consent for research was obtained from all participants in the study.

### Clinical and neuropsychological assessments

For each participant, the disease stage was measured using the HY state score, and the disease severity was assessed by the Movement Disorder Society-Unified Parkinson's Disease Rating Scale III (MDS-UPDRS III). All participants were administrated the University of Pennsylvania Smell Identification Test (UPSIT) for assessment of the olfactory function with lower scores indicating worse performance and with UPSIT score > 33 considering normal olfactory function [17]. Autonomic symptoms were assessed by Scales for Outcomes in Parkinson's disease - Autonomic (SCOPA-AUT) which covers six different autonomic domains including gastrointestinal, urinary, cardiovascular, thermo-regulatory, pupillomotor, and sexual with higher scores reflecting more severe autonomic dysfunction [18]. Depression was tested by 15-item Geriatric Depression Scale (GDS-15). Assessment of the neuropsychological state included MoCA for global cognition, Benton Judgment of Line Orientation Score (JoLO) for visuospatial ability, Hopkins Verbal Learning Test (HVLT) (immediate and delayed recall) for memory, Letter-Number Sequencing (LNS) and Semantic Fluency for executive function/working memory, and Symbol Digit Modalities Test (SDMT) for attention. Clinical characteristics and neuropsychological assessments were presented in Table 1.

**Figure 1.**
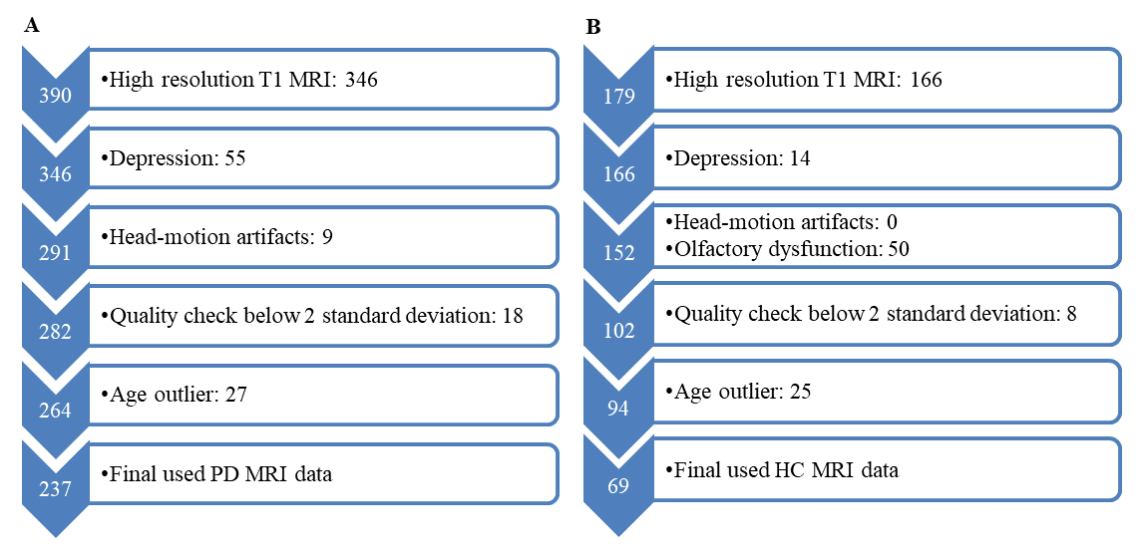
Flow chart of MRI data inclusion and quality control. (A) for PD; (B) for HC.

### MRI data inclusion and quality control

As shown in Figure 1, the quality control included the following steps: i) high resolution T1 MRI were included; ii) participants did not suffer from depression with GDS < 5 [19]; iii) participants without head-motion artifacts; iv) normalized GM data below 2 standard deviations were excluded after quality check; v) participants with age outliers were excluded due to the close association between aging and GM atrophy [20]. Besides, HCs who exhibit olfactory dysfunction measured by UPSIT [17] were further excluded. In total, 237 early, drug-naïve non-demented PD patients and 69 HCs remained.

### MRI data acquisition parameters

GE Medical Systems, Philips Medical Systems, and SIEMENS scanners were used for the MRI acquisition. T1 images of 44 subjects (10 using 1.5T and 34 using 3T) were obtained using GE Medical Systems with the following protocols: matrix x = 256, y = 256, z = 152-248, Flip angle: 8-15, TE = 3.02-5.17 ms, TR = 8.16-13 ms, slice thickness = 1.2-1.4 mm; 51 subjects (11 using 1.5T and 40 using 3T) using Philips Medical Systems with the following protocols: matrix x = 240-268, y = 192-256, z = 136-170, Flip angle = 8, TE = 3.16-4.01 ms, TR = 6.83-8.51 ms, slice thickness = 1-1.2 mm; and 211 subjects (20 using 1.5T and 191 using 3T) using SIEMENS with the following protocols: matrix x = 192-256, y = 192-256, z = 128-192, Flip angle = 8-15, TE = 2.27-3.65 ms, TR = 1900-2400 ms, slice thickness = 1 mm.

**Table 2:** Demographic and neuropsychological characteristics of the subsets of HCs and PD-NC matched in age, gender, and education with PD-MCI.

	HC (*n* = 61)	PD-NC (*n* = 61)	PD-MCI (*n* = 61)	*p*-value
Age (year)	62.21 (6.43)	61.80 (7.70)	64.11 (7.08)	0.194
Gender (m/f)	36/25	35/26	37/24	0.934
Education (year)	16.16 (2.25)	15.26 (3.38)	14.93 (3.14)	0.059
TIV	1534.55 (199.17)	1528.21 (139.93)	1559.40 (144.16)	0.117
HY	—	1.56 (0.50)	1.67 (0.51)	0.229
MDS-UPDRS Part III	—	19.11 (8.21	22.75 (9.28)	0.024
Disease duration (month)	—	7.03 (7.41)	7.03 (7.23)	0.555
JoLO	13.30 (1.63)	13.03 (2.06)	12.18 (2.22)	0.007^+^‡
GDS	0.90 (1.00)	1.39 (1.36)	1.74 (1.17)	<0.001†
MoCA	28.26 (1.09)	28.26 (1.26)	24.34 (2.11)	<0.001†‡
HVLT-R immediate recall^#^	26.26 (4.24)	24.64 (4.57)	21.56 (5.29)	<0.001†‡
HVLT-R delayed recall	9.38 (2.27)	8.64 (2.35)	7.20 (2.54)	<0.001†‡
LNS	10.72 (2.23)	11.21 (2.71)	9.49 (2.59)	0.001†‡
Semantic fluency total	54.13 (10.86)	48.85 (10.49)	44.26 (10.38)	<0.001†
SDMT	48.69 (9.68)	44.31 (9.92)	36.90 (11.38)	<0.001†‡
UPSIT	36.74 (1.63)	24.02 (8.51)	20.33 (7.82)	<0.001*†‡
SCOPA-AUT	5.21 ± 2.60	7.90 ± 3.80	10.38 ± 6.07	<0.001*†‡

Note: Data are expressed as mean (standard deviation). Gender data were analyzed with χ^2^ test. Other *p* values were derived from Kruskal Wallis test or Mann-Whitney test for non-parametric test and independent one-way ANOVA or two sample *t*-test for parametric-test.

* post hoc comparisons showed significant differences between HCs and patients with PD-NC;

† post hoc comparisons showed significant differences between HCs and patients with PD-MCI;

‡ post hoc comparisons showed significant differences between patients with PD-NC and those with PD-MCI. TIV = total intracranial volume; MDS-UPDRS = Movement Disorder Society-Unified Parkinson's Disease Rating Scale; JoLO = Benton Judgement of Line Orientation; GDS = Geriatric Depression Scale; MoCA = Montreal Cognitive Assessment; LNS = Letter-Number Sequencing; HVLT-R = Hopkins Verbal Learning Test-Revised; SDMT = Symbol-Digit Modalities Test; UPSIT = University of Pennsylvania Smell Identification Test; SCOPA-AUT = Scales for Outcomes in Parkinson's disease - Autonomic; HC, healthy control; PD-NC, Parkinson's disease with normal cognition; PD-MCI, Parkinson's disease with mild cognitive impairment.

### Definition of MCI in PD

In the present study, MCI in PD was defined according to the MDS level I guideline with MoCA score less than 26 and more than 21 [21,22]. Besides, participants in the current study were also classified as PD-MCI if they scored at least 1.5 SD below the standardized mean score on two or more neuropsychological tests [22]. Thus, among the remaining 237 PD patients, 61 PD were classified as PD-MCI in which 20 subjects had cognitive decline reported by either the subject or the informant, or clinically interviewed by the clinician. To further reduce the confounding effect of different sample size on GM volume changes among groups, two subsets of 61 HCs and 61 PD-NC who were closely matched to 61 PD-MCI for age, gender, and education level (subject by subject) were included (see Table 2).

### VBM-DARTEL analysis

To determine the structural abnormalities in early PD-MCI patients, we performed a VBM analysis for all structural images of PD-NC, PD-MCI, and HC using Cat12 (Gaser C, Jena University Hospital, http://dbm.neuro.uni-jena.de/cat/) and SPM 12 (Statistical Parametric Mapping, Wellcome Department of Imaging Neuroscience, London, UK). All T1-weighted images were spatially normalized using the Diffeomorphic Anatomical Registration using Exponentiated Lie algebra (DARTEL) algorithm [23] and segmented into gray matter (GM), white matter (WM) and cerebrospinal fluid (CSF) [24]. Non-linear warping of GM images was then performed to the GM template in MNI space. The Spatially normalized GM maps were modulated by the Jacobian determinant of the deformation field and corrected for individual brain sizes. The modulated, normalized GM images (voxel size 1.5×1.5×1.5 mm^3^) were smoothed with an 8-mm full width at half maximum isotropic Gaussian kernel. The total intracranial volume (TIV) was represented by the sum of the GM, WM, and CSF volumes.

### Statistical analysis

Normality of clinical and neuropsychological data was firstly evaluated by Kolmogorov-Smirnov (KS) test in order to choose appropriate parametric and non-parametric tests using SPSS v22. Independent one-way analysis of variance (ANOVA) (for parametric test) or Kruskal-Wallis test (for non-parametric test) was performed for comparing the three groups, with Bonferroni correction for the post-hoc comparisons. Significance was determined by *p* < 0.05.

The GM maps were analyzed using general linear model on a voxel-wise comparison across the whole brain, and two models were designed and employed. First, regionally differences in GM volume among the three groups were assessed using an analysis of covariance (ANCOVA) with age, gender, education level, TIV, and GDS score as nuisance variables. Post-hoc comparisons between groups were conducted by two-sample *t*-test. A conjunction analysis was performed on the contrast of (HC > PD-NC) and (HC >PD-MCI) to detect the GM volume atrophy common to PD-MCI and PD-NC. Besides, another model of the subset groups with age, gender and education level matched was designed to eliminate the confounding effect of sample size on GM changes among groups. ANCOVA results were reported based on an uncorrected voxel-wise height threshold of *p* < 0.001 combined with an FWE-corrected cluster-wise threshold of *p* < 0.05. Results of post-hoc comparisons were thresholded with an uncorrected voxel-wise threshold of *p* < 0.001. Then due to our apriori hypothesis about the GM alteration, a small volume correction (SVC) was applied to investigate which subregions in the MTL might be more affected in PD-MCI patients and results were thresholded at FWE-corrected *p* < 0.05. Brain regions were localized based-on Anatomy toolbox v2.2c [25].

### Correlation analysis

Region-of-interest (ROI) analysis was performed on the regions that showed significant GM changes that are common to the two PD groups and regions that are specific to PD-MCI. These ROIs were defined based on the corresponding identified clusters. GM volumes in each ROI were computed by the sum of each voxel's volume in the ROI. To explore the correlation between GM volume and performance on neuropsychological assessments and non-cognitive assessments, partial correlations were then performed, controlled for age, gender, education level, TIV, and GDS score. In addition, receiver operating characteristic (ROC) curve was applied to assess the discriminant performance of brain regions in these ROIs to distinguish PD-MCI from PD-NC.

## RESULTS

### Demographic and neuropsychological results

As shown in Table 1, no significant difference was found in age, gender, and TIV among three groups. Significant higher education level was found in HC compared to PD-NC (*p* < 0.05) and PD-MCI (*p* < 0.05). No significant difference was found in disease duration and HY scores between PD-NC and PD-MCI, while PD-MCI patients showed significantly higher MDS-UPDRS score (*p* < 0.005) compared to PD-NC.

Compared to HCs, PD-NC patients showed significant deficits in GDS (*p* < 0.005), USPIT (*p* < 0.001), SCOPA-AUT (*p* < 0.001), and the cognitive domain of attention measured by SDMT (*p* < 0.001). Besides above-mentioned dysfunction, PD-MCI patients additionally exhibited deficits in all cognitive domains compared to HCs (Table 1). Furthermore, comparing to PD-NC, PD-MCI showed significant cognitive impairments in MoCA (*p* < 0.001), JoLO (*p* < 0.005), Semantic Fluency (*p* < 0.001), HVLT Immediate and Delayed Recall (*p* < 001), SDMT (*p* < 0.001), as well as LNS (*p* < 0.001). Among these cognitive tests, the most common domain affected in PD-MCI was attention measured by SDMT (Cohen’s d = 1.15) compared to HC; while compared to PD-NC, the most severe domain affected in PD-MCI was memory measured by HVLT-R immediate recall (Cohen’s d = 0.81). In the non-cognitive assessments, PD-MCI exhibited specifically dysfunction in UPSIT (*p* < 0.05) and SCOPA-AUT (*p* < 0.05) with gastrointestinal domain most impaired in PD-MCI compared to HC (Cohen’s d = 1.18).

**Figure 2.**
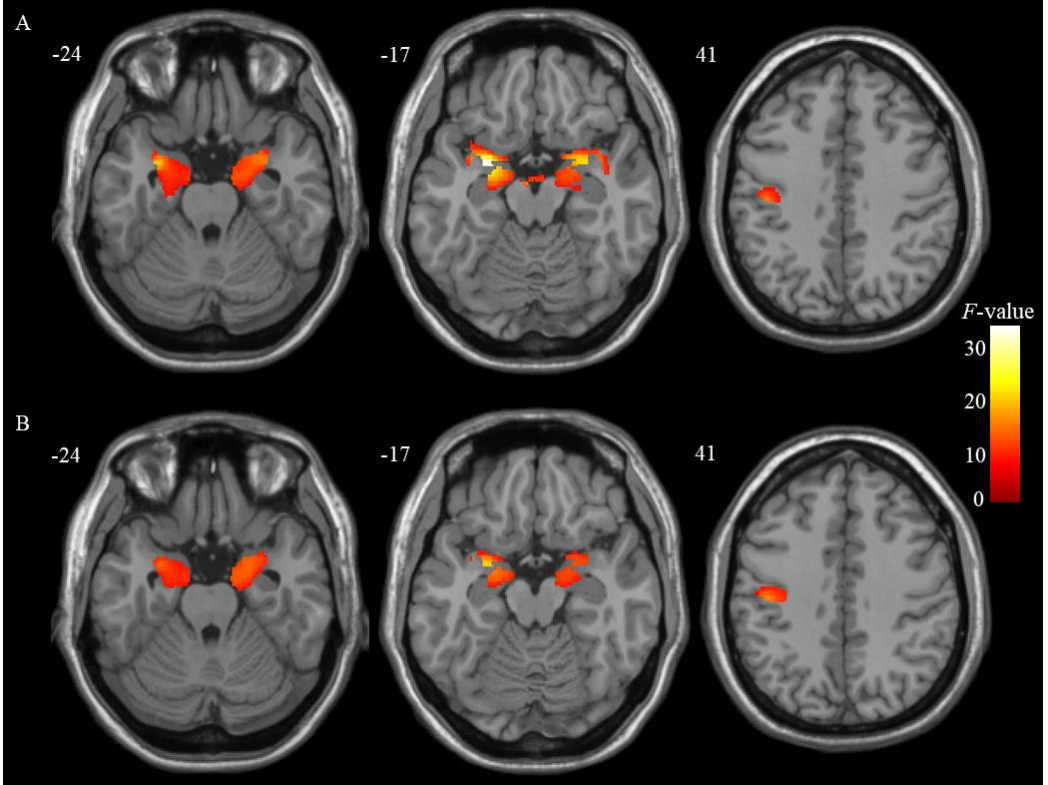
ANCOVA results of GM alterations among the three groups. (A) for all subjects; (B) for the subsets of groups with sample size matched.

### VBM results

The ANCOVA analysis revealed significant bilateral medial temporal atrophies in the amygdala (AM) and the left primary motor cortex (M1) among the three groups controlled for age, gender, education level, TIV, and GDS score (see Table 3 and Fig. 2A).

GM volume changes in PD-NC and PD-MCI.

**Figure 3.**
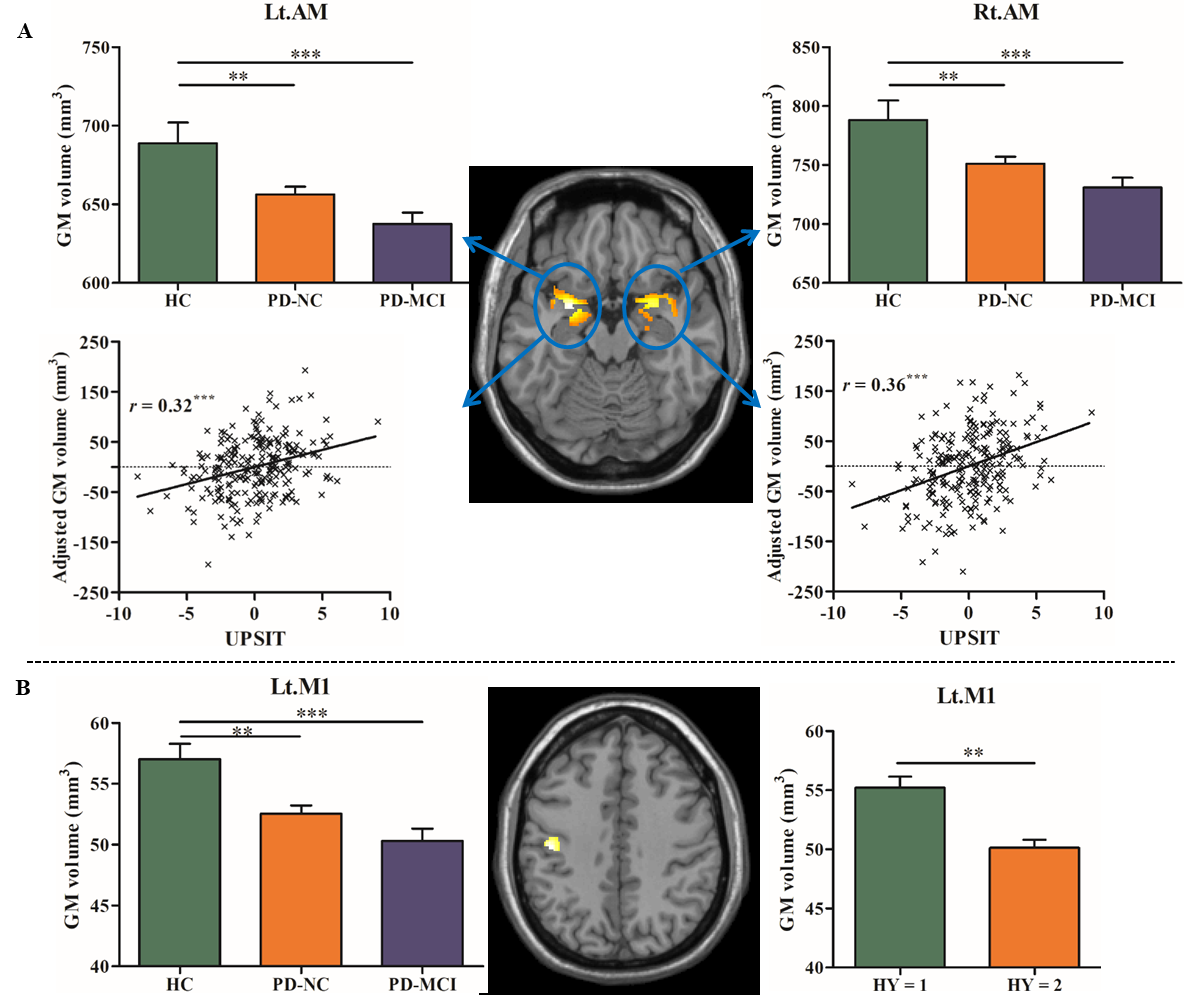
GM atrophy in the bilateral amygdala (AM) (A) and left primary motor cortex (M1) (B) common to PD-NC and PD-MCI. The bar charts show the mean GM volume in each ROI. The scatterplots indicate the positive correlation between volumes in the bilateral AM and olfactory function measured by UPSIT adjusted for age, gender, education level, TIV and GDS score in each ROI in combined PD patients. ^**^ represents *p* < 0.01 and ^***^ represents *p* < 0.001.

Compared to HC, GM atrophies in the bilaterally AM and the left M1 were commonly identified in both PD-NC and PD-MCI groups (see Table 3 and Fig. 3).

Comparisons of GM volume between PD-NC and PD-MCI.

**Figure 4.**
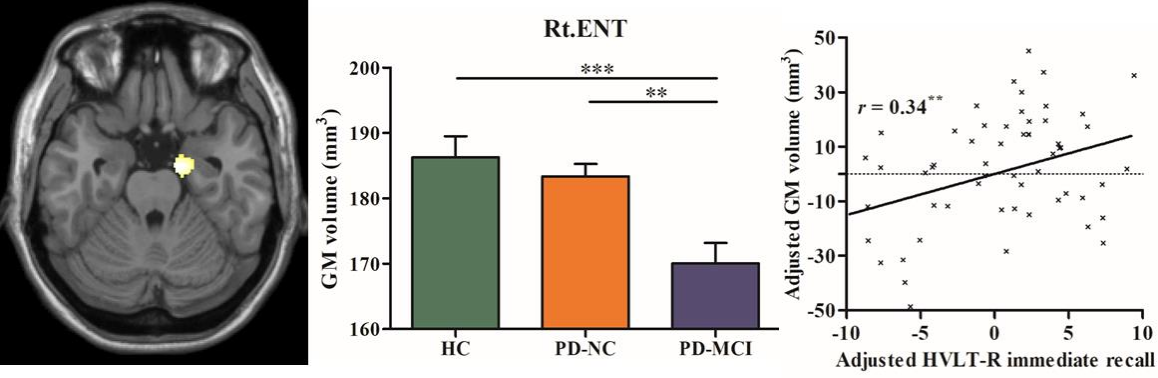
Right entorhinal cortex (ENT) atrophy specific to PD-MCI. The bar charts show the mean GM volume in the ENT. The scatterplots indicate the positive correlation between ENT volumes and memory performance measured by HVLT-immediate recall adjusted for age, gender, education level, TIV, and GDS score in PD-MCI patients. ^**^, represents *p* < 0.01.

In contrast to PD-NC, the right entorhinal cortex (ENT) atrophy was specifically detected in PD-MCI (see Table 3 and Fig. 4).

Subsequently, these regions including the bilateral AM and left M1 that exhibited atrophies common to both PD groups and the right ENT that showed atrophy specific to PD-MCI were further defined as regions of interest (ROIs). In addition, the subsets of the three groups exhibited very similar results to the whole groups, which further confirmed these findings ruling out the confounding effects of different sample sizes (see Table 4 and Fig. 2B).

### Correlation results

The correlation analysis results showed that significantly positive correlations were observed between the olfactory performance (measured by UPSIT test) and the bilateral AM volumes (*r* = 0.32 and *p* < 0.001 for left side; *r* = 0.36 and *p* < 0.001, respectively) in combined PD patients (see Fig. 3A). The comparison results of volume differences showed that patients with PD with HY = 2 exhibited smaller M1 volume compared to those patients with HY = 1 (see Fig. 3B). Moreover, positive correlation between memory performance (measured by HVLT-immediate recall) and the volumes in the right ENT was specifically revealed in PD-MCI (*r* = 0.34, *p* < 0.01) (see Fig. 4). No other significant correlation was detected in the other cognitive and non-cognitive tests. Furthermore, it was found that the areas under the curve (AUC) of ROC of the ENT volume (0.70) was higher than that of the AM (0.58) and M1 (0.56) to discriminate PD-MCI from PD-NC.

## DISCUSSION

The present study aimed to detect neuroanatomical alterations that are specific to early, drug-naive PD-MCI in contrast to early, drug-naive PD-NC and HC. It was found that the brain volumes of bilateral AM and the left M1 were commonly reduced in both PD groups; while the right ENT atrophy was found specific to PD-MCI, which might help to discriminate MCI from non-demented PD.

**Table 3:** Clusters of significant gray matter alterations among the three groups.

Anatomical regions	Cluster size (voxel)	MNI (x, y, z)	*F*/*T*-value
**ANCOVA**			
Lt.AM	2095	-27, 0, -17	32.17
Rt.AM	3761	29, 3, -17	19.41
Lt.M1	364	-44, -20, 41	13.70
**HC > PD-NC**			
Lt.AM	1534	-29, 0, -17	7.37
Rt.AM	2063	30, 3, -17	5.77
Lt.M1	364	-42, -21, 41	5.08
**HC > PD-MCI**			
Lt.AM	1798	-26, 0, -17	7.10
Rt.AM	2278	26, 3, -18	5.64
Lt.M1	168	-45, -18, 41	4.36
**Conjunction**			
Lt.AM	1237	-27, 0, -17	7.07
Rt.AM	1483	29, 3, -18	5.57
Lt.M1	168	-45, -18, 41	4.36
**PD-NC > PD-MCI**			
Rt.ENT	315	15, -9, -24	4.02

Note: AM = amygdala; ENT = entorhinal cortex; M1 = primary motor cortex; Conjunction refers to (HC > PD-NC) in conjunction with (HC > PD-MCI); Lt = left; Rt = right; HC =healthy control; PD-NC = Parkinson’s disease with normal cognition; PD-MCI =Parkinson’s disease with mild cognitive impairment. Results were thresholded by using a voxel height threshold *p* < 0.001 and cluster-corrected by using family-wise error (FWE) *p* < 0.05 or small volume correction (SVC) p < 0.05 FWE-corrected.

### GM volume atrophies common to PD groups

In the present study, PD patients exhibited significantly smaller AM compared to HCs, which supports the previous findings in this field [4,6,10,11]. In particular, the AM is considered to be an important structure in olfaction processing [26] due to its interconnections with entorhinal cortex and hippocampus [27]. Olfactory deficits appear early in the course of PD. In the present study, positive correlations were found between the AM atrophy and the olfactory impairment in PD patients. Thus, the pattern of the results might suggest that the olfactory impairment of the disease contributes to the AM atrophy in PD. For example, Wattendorf *et al*. (2009) [11] also reported the significant amygdala atrophy as well as its correlation with olfactory performance in PD patients. This finding may indicate that the AM is particularly vulnerable to degeneration in PD patients, which is independent of cognitive state even at early stage.

The dopaminergic balance within the motor loop is disrupted in individuals with PD due to the depletion of dopamine in the striatum [28], which in turn impairs its projection to motor cortex, especially to M1 [29]. Animal model of PD has pointed out that the dopamine deletion leads to altered oscillatory activities and synchronization of local field potentials in M1 [30]. Numerous brain stimulation and neuroimaging studies have demonstrated the pivotal role of M1 in motor performance [31,32]. In the present study, it was found that patients with PD exhibited significant M1 atrophy compared to HCs. The pattern of results seemed to suggest that the reduction of the M1 volume is closely related to the severity of PD disease.

### Atrophy in the right ENT specific to PD-MCI

It is noteworthy that, the finding of ENT atrophy was specifically detected in PD-MCI which differentiated individuals with PD-MCI from cognitively normal PD. Previous studies in MCI and early Alzheimer’s disease (AD) have demonstrated that the ENT is involved very early in disease-related pathology, even before changes in the hippocampus [33]. The ENT plays a crucial role in memory processing [34,35,36] as it acts as a gateway to project to fields of hippocampal formation [37,38]. In the present study, the most affected domain in PD-MCI was memory measured by HVLT-R immediate recall (Cohen’s d = 0.81) in compared to PD-NC. Accordingly, the ENT volume atrophy is positively associated with reduced memory performance in PD-MCI. Consistent with previous study [39], the finding of ENT atrophy specific to PD-MCI which further associated with memory decline in this disease, may suggest a dementia-related pathology in this disease.

The potential prediction of the hippocampus in the development of cognitive impairment in PD has been reported previously [40] and the hippocampal volume loss has been implicated in PD-MCI patients when compared to PD-NC in previous studies [4,41]. However, in the present study, no significant hippocampus loss was specifically detected in PD-MCI when compared with PD-NC. Previous pathological studies have suggested that neuropathological hallmarks first accumulate in the ENT and then in the hippocampus [42,43]. Along with this line, the heterogeneity of PD patients might account for this discrepancy, that is, the early, untreated PD patients in the present study vs. the moderate or advanced, treated PD patients in previous studies [4,41]. In addition, the different data processing method may also contribute to the discrepancy to some extent, for example, the voxel-based whole brain analysis in the present study vs. ROI-based measurement which may induce bias of ROI selection in previous studies [4,41]. Finally, aging-related hippocampus atrophy would be more pronounced in patients with PD-MCI relative to PD-NC [41]. In the previous study [4], the PD-MCI patients were significantly older than PD-NC (*p* = 0.01), which may confound additional aging-related hippocampus atrophy processes in PD-MCI.

It should be mentioned that in the present study the drug-naive PD-MCI patients are different from those under L-dopa treatment. By relieving motor symptoms in PD, the use of L-dopa might exert an effect on certain aspects of cognition such as executive function, learning, and memory [44,45]. However, dopamine treatment might ameliorate cognitive function in some cases [46] but might also impair it in some other conditions (dopamine overdose) [47]. The different severity of dopamine depletion may explain the heterogeneous behavioral outcome. Particularly, in early stage of the disease with mild dopamine deficit, the L-dopa treatment of motor dysfunction may result in overdose in cortico-striatal circuit [48]. Together, treated or untreated PD-MCI patients may be one of the confounding effects which may affect the group difference between PD-NC and PD-MCI.

**Table 4:** Clusters of significant GM alterations among the subsets of groups.

Anatomical regions	Cluster size (voxel)	MNI (x, y, z)	*F*/*T*-value
**ANCOVA**			
Lt.AM	1266	-27, 2, -17	18.78
Rt.AM	1103	23, 2, -24	12.32
Lt.M1	499	-44, -20, 41	14.73
**HC > PD-NC**			
Lt.AM	497	-29, 2, -17	4.49
Lt.M1	363	-42, -20, 39	4.75
Rt.AM^#^	73	30, 3, -17	3.87
**HC > PD-mci**			
Lt.AM	1239	-27, 2, -17	6.00
Rt.AM	1103	23, 3, -24	4.91
Lt.M1	430	-44, -20, 42	4.72
**Conjunction**			
Lt.AM	340	-29, 2, -17	4.20
Lt.M1	418	-44, -20, 39	4.68
Rt.AM^#^	73	30, 3, -17	3.06
**PD-NC > PD-MCI**			
Rt.ENT	385	12, -6, -24	4.05

Note: AM = amygdala; ENT = entorhinal cortex; M1= primary motor cortex; Lt = left; Rt = right; HC =healthy control; PD-NC = Parkinson’s disease with normal cognition; PD-MCI =Parkinson’s disease with mild cognitive impairment. Results were thresholded by using a voxel height threshold *p* < 0.001 and cluster-corrected by using family-wise error (FWE) *p* < 0.05 or small volume correction (SVC) *p* < 0.05 FWE-corrected except for ^#^ which is derived from voxel-wise *p* < 0.005 uncorrected.

In conclusion, the current study suggests that the right ENT atrophy may be a potential neuroanatomical biomarker specific to early, drug-naive PD-MCI, which could possibly be used as surrogate endpoints to assess and monitor the efficacy of pharmacological interventions in the future. The findings also shed light on the neural underpinnings of the disease and provide information on the different states of PD (e.g. PD-MCI and PD-NC). However, several limitations should be recognized. First, it could not exclude the influence of AD-like pathology due to a lack of autopsy proven data. Second, the present study employed the MDS Level I criteria to classify PD-MCI patients, which could not justify the underlying neurobiological substrates and clinical courses in different cognitive domains. Finally, to fully understand the mechanism of the disease, further longitudinal studies considering the patients who evolved to dementia will need to be examined.
